# Overcoming immune resistance in advanced esophageal squamous cell carcinoma with recombinant human adenovirus type 5 by impacting the immune microenvironment: a case report

**DOI:** 10.3389/fimmu.2025.1610058

**Published:** 2025-06-30

**Authors:** Zhongting Wang, Junfeng Hong, Huiping Wu, Yan Zheng, Xinchen Lin, Ying Lin, Xuzhou Wang, Wenzheng Fang

**Affiliations:** ^1^ Department of Oncology, The Affiliated People’s Hospital of Fujian University of Traditional Chinese Medicine, Fuzhou, Fujian, China; ^2^ Department of Oncology, The 900th Hospital of PLA Joint Logistic Support Force (Fuzong Clinical Medical College of Fujian Medical University), Fuzhou, Fujian, China; ^3^ Department of Ultrasound, The 900th Hospital of PLA Joint Logistic Support Force (Fuzong Clinical Medical College of Fujian Medical University), Fuzhou, Fujian, China; ^4^ Department of Intervention, The Affiliated People’s Hospital of Fujian University of Traditional Chinese Medicine, Fuzhou, Fujian, China; ^5^ Department of Pathology, The 900th Hospital of PLA Joint Logistic Support Force (Fuzong Clinical Medical College of Fujian Medical University), Fuzhou, Fujian, China

**Keywords:** ESCC, H101, immune resistance, intralymphatic administration, case report

## Abstract

Advanced esophageal squamous cell carcinoma (ESCC) has a poor prognosis. Chemotherapy combined with immune checkpoint inhibitors is a feasible treatment, but effective treatment modalities need to be explored for its immune resistance. H101, a genetically modified oncolytic adenovirus, represents a promising anti-tumor therapeutic strategy due to its ability to selectively replicate in and lyse cancer cells while sparing normal tissues. H101 has shown clinical efficacy in treating nasopharyngeal carcinoma and hepatocellular carcinoma. However, its therapeutic potential in ESCC remains understudied, with limited reports available. We reported a case of a patient with multiple relapses of advanced ESCC who exhibited a progression-free survival (PFS) of 15.5 months following the administration of first-line chemotherapy in conjunction with immunotherapy. At first recurrence, the patient received H101 injection in metastatic lymph nodes with chemo- and immunotherapy, demonstrating a reduction in the left cervical lymph node from 29.1×12.9 mm to 24.6×10.36 mm at 25 days post-injection, and ultimately achieving a PFS of 30 months. During the second recurrence, after undergoing three cycles of the aforementioned combined treatment regimen, the patient experienced significant alleviation of their disease. Following the H101 injection, we noted that the patient experienced transient fever, lymph nodes at both injection and non-injection sites subsided, pathological complete response was achieved, and PFS was significantly prolonged. We also observed significant increases in the expression of CD3, CD4, CD8, CD20 and IL-1β after two relapsed H101 treatments based on multiplex immunofluorescence analysis. Intralymphatic injection of H101 combined with chemotherapy and immunotherapy may represent a promising clinical strategy for advanced ESCC patients with recurrent lymph node metastasis.

## 1 Introduction

Esophageal cancer (EC) represents a significant global health burden, ranking as the seventh most commonly diagnosed malignancy and the sixth leading cause of cancer-related mortality worldwide in 2020, with more than 50% of global cases occurring in China (1). EC is pathologically classified into two main subtypes: esophageal squamous cell carcinoma (ESCC) and esophageal adenocarcinoma. Among these, ESCC predominates significantly, representing over 90% of all EC cases annually (2). Notably, ESCC is frequently diagnosed at a locally advanced stage or with metastasis, resulting in a poor prognosis with a five-year survival rate ranging from 10% to 25% (1). Immune checkpoint inhibitors (ICIs) combined with chemotherapy have become the first-line treatment standard for advanced unresectable ESCC, offering the potential for improved long-term survival in certain patients with this disease (3). However, the majority of patients remain at risk of drug resistance and relapse, so studying and overcoming drug resistance is crucial to enhancing the survival benefit of patients.

Oncolytic viruses (OVs), as a novel form of anti-tumor immunotherapy, possesses the ability to selectively replicate within tumor cells and directly lyse them (4). Study has demonstrated that the combination of OVs with other systemic therapies, including chemotherapy, immunotherapy, and targeted therapy, significantly enhances therapeutic efficacy (5). This is primarily due to its ability to recruit immune cells to the tumor microenvironment, thereby overcoming tumor-mediated immune evasion mechanisms and potentiating anti-tumor immune responses (5). Recombinant human adenovirus type 5 (H101), a genetically engineered oncolytic adenovirus, was approved by the China National Drug Administration in 2005 for the treatment of nasopharyngeal carcinoma (6). H101 was constructed using genetic recombination technology, based on an adenovirus type 5 (Ad5) backbone, with deletions in the E1B-55kD gene and partial E3 region (7). Clinical studies have demonstrated that intraperitoneal injection of H101, with or without chemotherapy, can significantly reduce malignant pleural effusion and ascites caused by lung cancer or gastrointestinal tumors (8). In addition, intratumoral injection of H101 combined with chemotherapy confirmed efficacy in alleviating local lesions associated with advanced gastric carcinoma and cervical cancer (9, 10). Importantly, the combination of H101 and ICIs has revealed significant therapeutic efficacy in overcoming immune resistance both in small cell lung cancer and non-small cell lung cancer (11, 12). Although OVs may sensitize “cold” tumors to ICIs by modulating the tumor immune microenvironment, there is still a lack of substantial clinical data indicating the efficacy and safety of H101 combined with ICIs in patients with solid tumors. Currently, H101 is predominantly administered intratumorally, followed by intravenous drip and intraperitoneal administration, while intralymphatic administration remains uncommon.

Here, we report a patient with stage IV ESCC who relapsed after chemotherapy combined with immunotherapy. Following the administration of metastatic lymph node injection with H101, in conjunction with continued chemotherapy and immunotherapy, both the injected and non-injected lymph node sites showed remarkable shrinkage, and pathologic complete response (pCR) was achieved, and progression free survival (PFS) reached 30 months. Upon the occurrence of a second recurrence, given the patient’s demonstrated high sensitivity to OVs therapy, the same treatment regimen was re-administered. After three cycles of treatment, the disease showed substantial remission once again. Additionally, we investigated the potential molecular mechanisms underlying the efficacy of H101 against ESCC.

## 2 Case presentation

**Figure 1:**
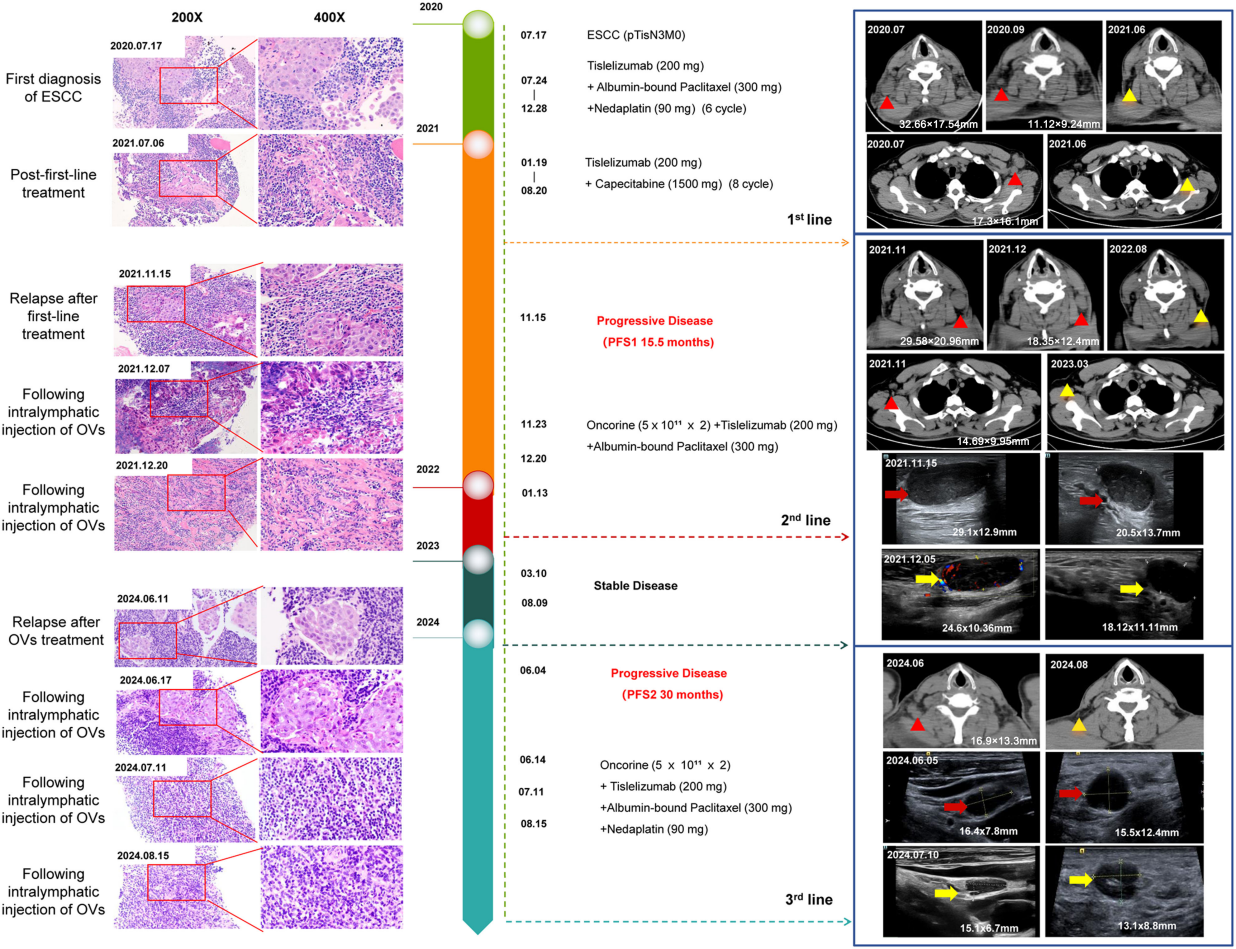
Timeline of the disease progression and relevant treatments of the patient. Hematoxylin-eosin (HE) staining of an esophageal mucosal biopsy specimen was shown on the left; The CT and color ultrasound images of the neck were shown on the right. 2020.07: At the first diagnosis of ESCC, axillary lymph node metastases showed a pathological phenotype of immune rejection; computed tomography scan showed right cervical and left axillary lymphadenopathy. 2021.06: After first-line treatment, no tumor cells were observed in lymph node metastases, achieving pathological complete remission; regression of previously enlarged lymph nodes. 2021.11: Recurrence after first-line treatment, cervical lymph node metastases showed a pathological phenotype of immune rejection; computed tomography scan showed left cervical and right axillary lymphadenopathy. 2021.11-2021.12: Doppler ultrasound of the left cervical lymph nodes showed that the two enlarged lymph nodes gradually shrank after H101 injection in the lymph nodes (One lymph node shrank from 29.1×12.9 mm to 24.6×10.36 mm, and the other shrank from 20.5×13.7 mm to 18.12×11.11 mm). 2024.06: Recurrence after H101 treatment, cervical lymph node metastases showed a pathological phenotype of immune rejection; computed tomography scan showed right cervical lymphadenopathy. 2024.06-2024.07: Doppler ultrasound of the right cervical lymph nodes showed that the two enlarged lymph nodes gradually shrank after H101 injection in the lymph nodes (One lymph node shrank from 16.4×7.8mm to 15.1×6.7 mm, and the other shrank from 15.5×12.4 mm to 13.1×8.8 mm).

In July 2020, an elderly man presented with painless, progressive enlargement of the right cervical lymph nodes. A PET-CT scan revealed multiple enlarged lymph nodes throughout the body, which were hypermetabolic and considered malignant (**Supplementary Figure 1**). We performed needle biopsy of axillary lymph nodes and pathology revealed squamous cell carcinoma (**Figure 1**). To identify the primary tumor lesion, the patient underwent electronic gastroscopy, which revealed a microscopic esophageal mass. Biopsy pathology of the esophageal mass indicated high-grade dysplasia of the squamous epithelium, suggesting ESCC, classified as stage IVa (pTisN3M0) according to the 2023 CSCO esophageal diagnosis and treatment guidelines. The patient presented with mild esophageal lesions accompanied by multiple systemic lymph node metastases, indicating advanced ESCC with no surgical indications. He received tislelizumab combined with nab-paclitaxel and nedaplatin, followed by tislelizumab combined with capecitabine as first-line treatment for 1 year. Throughout this period, multiple reexaminations of cervicothoracic computed tomography (CT) showed gradual regression of cervical lymph nodes (pre-treatment, 32.66×17.54 mm; two months, post-treatment11.12×9.24 mm) and no significant localized thickening of the esophageal wall.

**Figure 2:**
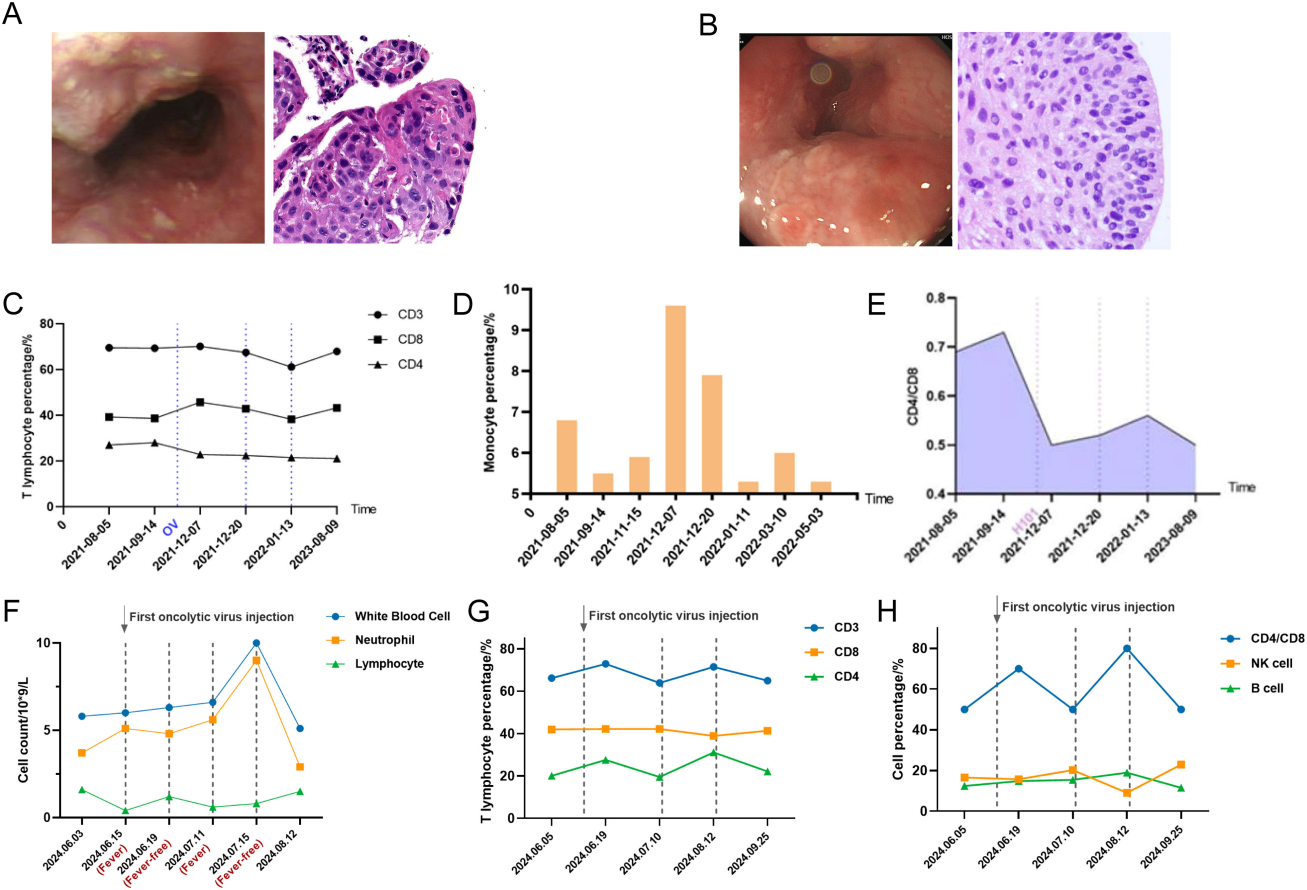
**(A)** The first recurrence of ESCC after first-line treatment as shown by electronic gastroscopy and pathological biopsy images. **(B)** Recurrence of ESCC after OVs injection as shown by electronic gastroscopy and pathological biopsy images. **(C)** Detection of peripheral blood T lymphocyte subsets after H101 injection for the first recurrence using flow cytometry (dashed lines indicate injection time points). **(D)** Detection of monocytes after H101 injection for the first recurrence showed an increase in monocytes. **(E)** Detection of peripheral blood CD4/CD8 ratio after H101 injection for the first recurrence (dashed lines indicate injection time points). **(F)** Peripheral blood parameter surveillance throughout febrile and remission phases after H101 injection for the secondary recurrence (dashed lines indicate injection time points). **(G)** Detection of peripheral blood T cell subsets after H101 injection for the secondary recurrence revealed an increase in CD3^+^ and CD4^+^ T lymphocytes (dashed lines indicate injection time points). **(H)** Detection of peripheral blood NK and B cell subsets after H101 injection for the secondary recurrence (dashed lines indicate injection time points).

In November 2021, the patient exhibited two newly enlarged lymph nodes (29.1×12.9 mm, 20.5×13.7 mm) in the left neck during a reexamination of the cervical CT and color Doppler ultrasound. The puncture pathology revealed metastatic squamous cell carcinoma, while the right axillary lymph nodes were also significantly enlarged, considering tumor immune resistance recurrence (**Figures 1**, **2A**). On November 23, after communicating with the patient and his family, the patient received an intravenous drip of tislelizumab and nab-paclitaxel combined with an intralymphatic injection of H101. A total of 1.0×10^12^ viral particles were injected into two lesions every 3 weeks for a total of 3 cycles of combined treatment. In July 2022, tislelizumab maintenance immunotherapy was continued for 2 cycles and then discontinued. No further treatment was performed after that. The patient’s left cervical lymph nodes showed significant shrinkage 25 days after the injection of H101 (24.6×10.36 mm, 18.12×11.11 mm), and they basically disappeared 9 months later. Additionally, the axillary lymph nodes also regressed 15 months after the injection. Furthermore, we observed an increase in peripheral blood CD8^+^ T lymphocytes and monocytes following intralymphatic injection of H101 (38.6% *vs.* 45.7%), with significant infiltration of CD8^+^ T lymphocytes in cancer nests. The CD4^+^/CD8^+^ ratio fluctuated around 0.5, indicating a good prognosis (**Figures 2C–E**).

In June 2024, the patient experienced swelling in the lymph nodes on the right side of his neck, prompting a reevaluation through a whole-body PET-CT scan, which revealed multiple metastases in the lymph nodes located on the right neck, mediastinum, hepatic portal, and retroperitoneum (**Supplementary Figure 1**). Upon combining the results of electronic gastroscopy with esophageal mucosa biopsy, it was determined that there was high-grade dysplasia of the squamous epithelium (**Figure 2B**). Biopsy of the right cervical lymph node indicated highly differentiated squamous cell carcinoma in the lymphoid tissue. Given the strong likelihood of esophageal origin, immunohistochemical analysis was conducted, revealing that tumor cells CK-p (+), CK5/6 (+), P40 (+), Ki-67 (40%+), and the positive rate of PD-L1 in tumor cells was 90%. The PFS was 30 months, considering the recurrence of drug resistance. Taking into account the high sensitivity of the patient to oncolytic virus, the combination therapy of H101 was used again for this recurrence. On June 14, 2024, July 11, 2024, and August 15, 2024, the patients were directly injected with 1.0×10^12^ virus particles into lymph node metastases under ultrasound guidance and were treated with tislelizumab, nab-paclitaxel and nedaplatin. It was observed that patients experienced transient fever within 6–8 hours following the administration of H101 injection. During this fever, the primary hematological changes included a decrease in lymphocytes and an increase in neutrophils, which reversed upon the fever’s resolution (**Figure 2F**). Analysis of T lymphocyte subsets in peripheral blood using flow cytometry revealed a significant increase in CD3^+^ (66.2% *vs.* 72.9%, *P*<0.001) and CD4^+^ (20.1% *vs.* 27.6%, *P*<0.001) T lymphocytes post-H101 injection (**Figures 2G, H**). Notably, as of July 10, 2024, the patient’s neck lymph nodes had decreased in size from 15.5×12.4 mm to 13.1×8.8 mm, indicating an overall improvement in their general condition and a high quality of life.

**Figure 3:**
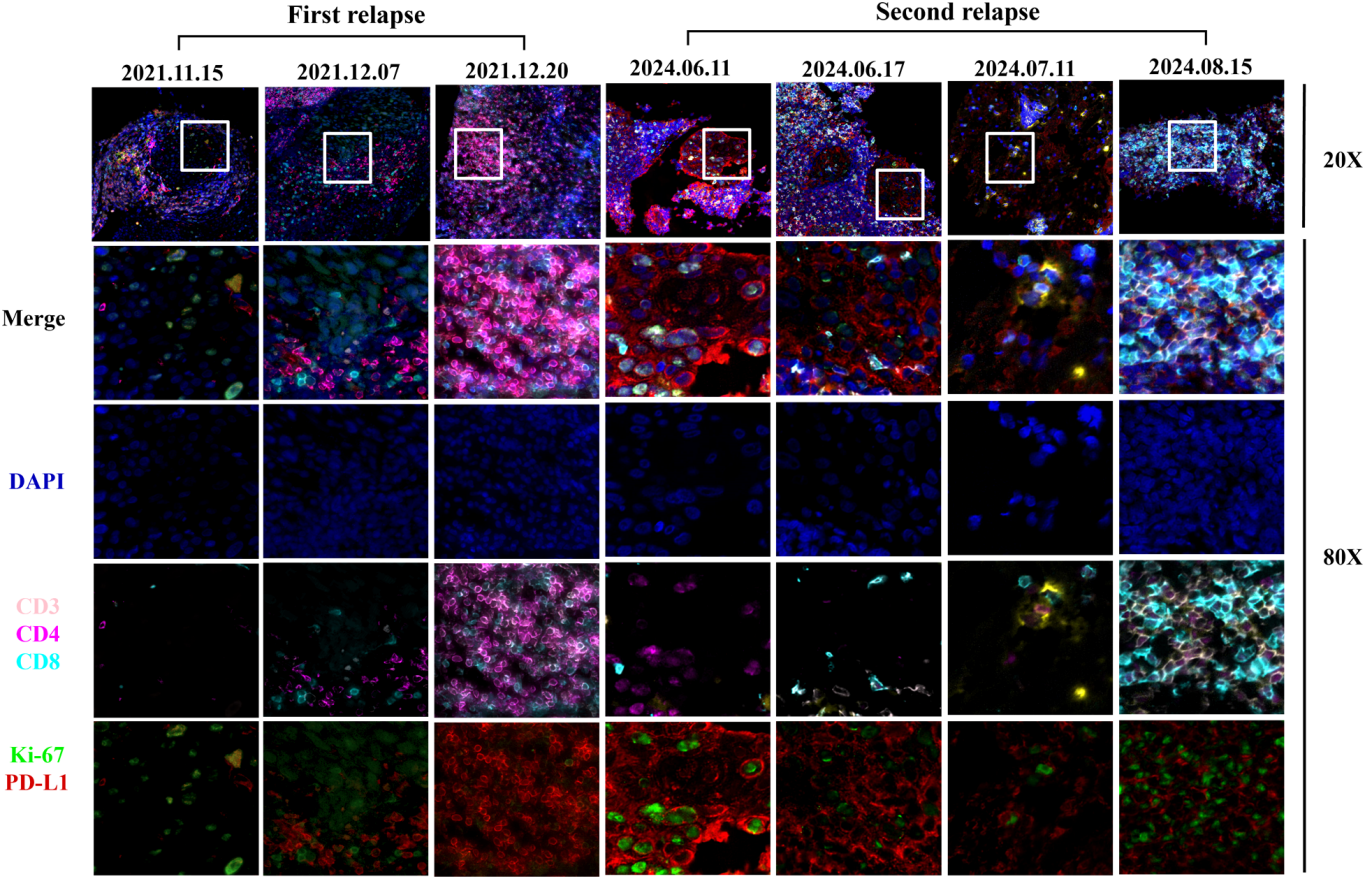
Images of multiple immunofluorescences staining after H101 injection for the first and second recurrence in cancer tissues. The three time points of the first recurrence in the figure represented before H101 injection, two weeks after H101 injection, and one month after H101 injection; The figure illustrated the dynamic changes in immune markers within tumor nests at four time points of the second recurrence: before H101 injection, 3 days after injection, 1 month after injection, and 2 months after injection. The results showed that after H101 treatment, the expression of CD3, CD4, CD8 increased within the tumor nests. Each recurrent biopsy was obtained from the same lymph node.

**Figure 4:**
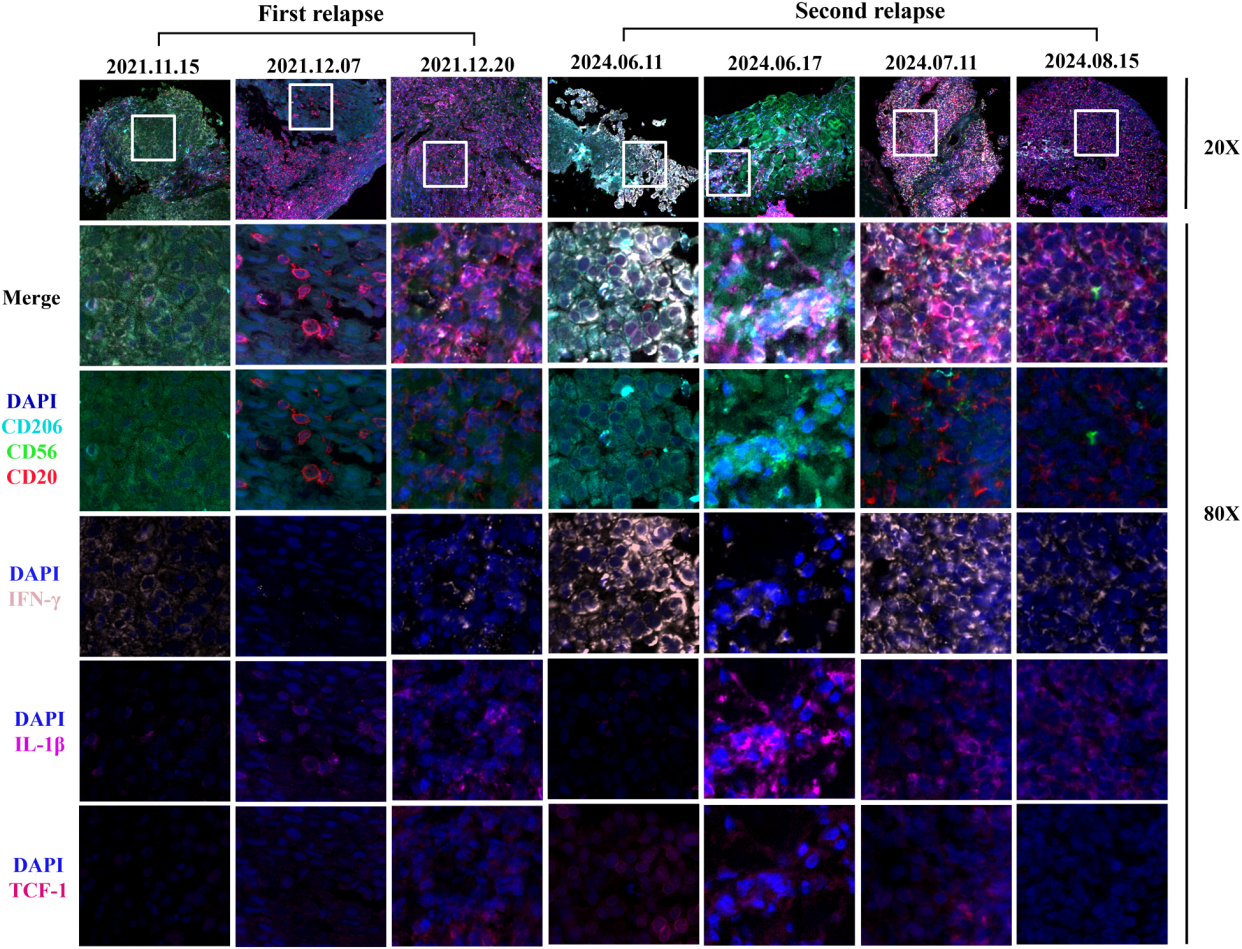
Images of multiple immunofluorescences staining after H101 injection for the first and second recurrence in cancer tissues. The three time points of the first recurrence in the figure represented before H101 injection, two weeks after H101 injection, and one month after H101 injection; The figure illustrated the dynamic changes in immune markers within tumor nests at four time points of the second recurrence: before H101 injection, 3 days after injection, 1 month after injection, and 2 months after injection. Following H101 treatment, we observed increased expression of CD20 and IL-1β. Notably, IFN-γ levels decreased at the two-week mark but rebounded by the one-month follow-up during the first recurrence. IFN-γ levels also showed a biphasic response during the second recurrence, with an initial decline at 3 days post-injection followed by recovery at 1 month. Each recurrent biopsy was obtained from the same lymph node.

Through hematoxylin-eosin (HE) staining of lymph nodes, we observed that the tumor immune microenvironment underwent a transition from immune rejection to immune cell infiltration two weeks after the initial H101 combination treatment, ultimately leading to pCR of lymph node metastases. Upon the second recurrence, the immune cells surrounding the cancer nest exhibited an immunoactivated phenotype infiltrating into the cancer nest 3 days after the treatment of H101 injected into the lymph node. Four weeks thereafter, a pCR of the lymph node metastasis was once again achieved. To further clarify the changes of tumor immune microenvironment, Pannoramic MIDI tissue imaging system (3DHISTECH) was used to perform multiple immunofluorescence analysis on pathological lymph node samples. We found that the expression of CD3, CD4, CD8, CD20, IL-1β, PD-L1 and TCF-1 in the cancer nest increased after H101 injection during the first recurrence (**Figures 3**, **4**; **Supplementary Figure 2**). In particular, the expression of IFN-γ decreased after 2 weeks of injection, and increased again after 1 month of injection. After the second recurrence, the expression of CD3, CD4, CD8, CD20 and IL-1β increased and the expression of PD-L1 and TCF-1 decreased in the cancer nest after H101 injection (**Figures 3**, **4**; **Supplementary Figure 2**). As with the first recurrence, IFN-γ expression decreased after 2 weeks of injection and increased after 1 month.

## 3 Discussion

To our knowledge, this is the first case report demonstrating the reversal of immune resistance in advanced ESCC by intralymphatic injection of H101 combined with ICIs. Currently, there is limited literature on the clinical utilization of OVs in the treatment of EC. A Phase I clinical trial conducted in Japan revealed that endoscopic intratumoral injection of OBP-301 combined with radiotherapy provided survival benefits for patients with esophageal cancer who were unfit for standard therapy (13). This offers a novel perspective for clinical diagnosis and treatment strategies. However, in this particular case, the primary esophageal lesion was not prominent, and the predominant manifestation was systemic lymph node metastasis, rendering the approach of intratumoral injection combined with localized radiotherapy evidently unsuitable. Study has demonstrated that there exists heterogeneity in the pathological response to immunotherapy between the primary tumor and its regional lymph node metastases (14). Lymph nodes, recognized as organs that enhance anti-tumor immunity, exhibit a more favorable response to immunotherapy. Specifically, metastatic lymph nodes display low heterogeneity and high levels of CD8^+^ T cell infiltration within the tumors (15). Therefore, we adopted a personalized treatment strategy for this patient, involving intralymph node metastasis injection of H101 combined with chemotherapy and immunotherapy, to achieve local and systemic anti-tumor effects. Although intralymphatic administration of H101 carries the risk of premature viral clearance due to host antiviral immunity, the pathological analysis of treated lymph nodes demonstrates that the direct oncolytic effects of H101, coupled with induced immune cell infiltration and immunogenic cell death, exert more potent therapeutic outcomes. In addition, we observed regression in axillary lymph nodes not injected with H101, which may be attributed to antigen cross-presentation.

We noted that fever was the only side effect present in terms of safety issues with H101 combination therapy, while it may also signify the degree of immune response mobilization in the patient. After injection of H101, the innate immune response system was initially activated, followed by the engagement of the adaptive immune system (16). This sequential activation resulted in an elevation in the number of neutrophils in the peripheral blood, accompanied by a decrease in lymphocyte count during fever, which is consistent with previous clinical study findings (13). After the first recurrence, the first injection of H101 led to an increase in CD8^+^ T cells in peripheral blood, indicating that the oncolytic virus directly lysed tumor cells, releasing a large number of tumor antigens and activating a CD8^+^ cytotoxic T cell response (17). After the second recurrence, the first injection of H101 resulted in little to no change in CD8^+^ T cells in peripheral blood, possibly due to T cell exhaustion. The shift from a decrease to an increase in CD4^+^ T cells may suggest a transition in the immune response from cytotoxicity-dominated to helper immunity. In addition, we found that CD4^+^ T lymphocytes were mainly elevated in peripheral blood at the first injection of H101 (+11%) after the second recurrence, which was contrary to the results of previous study (-17.2%) (18). However, study also indicated that wild-type oncolytic viruses may be effective against nasopharyngeal carcinoma by directly activating the helper CD4^+^ T cell response, possibly because different virus types have different changes in peripheral blood T lymphocyte subsets (19). It is worth noting that there was no particularly significant change in the percentage of T cells before and after H101 injection during each recurrence period. This may be because the effects of the OVs and PD-1 inhibitor primarily occur in the tumor microenvironment (TME) (20), while changes in peripheral blood may lag or be insignificant. However, this does not mean that no immune response is occurring within the tumor microenvironment. In lung cancer patients, there is no clear statistical correlation between changes in peripheral blood T-cell subsets and the efficacy of immunotherapy (21). Moreover, significant heterogeneity exists among different patients. In esophageal cancer patients receiving neoadjuvant chemotherapy and radiotherapy, changes in certain lymphocyte subsets in peripheral blood were associated with pathological complete response, but these variations differed substantially across individuals (22). Future studies should comprehensively integrate multiple immune indicators and changes in the tumor microenvironment to better evaluate treatment responses.

To investigate the alterations in the immune microenvironment subsequent to H101 injection, this study conducted an immunofluorescence analysis. The results showed that the expression levels of CD3, CD4, CD8, CD20 and IL-1β in the cancer nest were all increased after two recurrences, indicating that the immune cell infiltration was increased and the immune microenvironment was effectively activated (23). This finding is consistent with previous studies, indicating that OVs can further activate the immune response of T cells and B cells by directly lysing tumor cells and releasing tumor antigens (24–26). Notably, CD8^+^ T cells increased in both tumor and periphery after the first H101 injection, suggesting that H101 may systematically activate CD8^+^ T cells and promote their infiltration into tumors. In particular, we observed that during both relapses, IFN-γ levels exhibited a transient decrease at the initial phase of treatment (2 weeks), followed by a subsequent increase after one month of therapy. This dynamic alteration may be attributed to immunosuppressive mechanisms or immune cell exhaustion (27). The OVs could transiently suppress the TME during tumor cell lysis, potentially resulting in the temporary functional impairment of T cells and NK cells, consequently leading to a reduction in IFN-γ production. In addition, at the second recurrence, TCF-1 expression levels decreased, which was different from the results at the first recurrence. TCF-1 serves as a crucial transcription factor for the development and functional maintenance of T cells, and a reduction in its expression level may indicate T cell depletion or dysfunction (28). This disparity may be attributed to the dynamic alterations within the tumor microenvironment, particularly following multiple treatments, where tumor cells may evade immune surveillance through certain mechanisms, ultimately leading to the impairment of T cell function. Similarly, the expression trend of PD-L1 after two relapse treatments was consistent with that of TCF-1, which also reflected the dynamic changes of tumor microenvironment. During initial relapse treatment, tumor cells potentially upregulate PD-L1 expression as an adaptive response to enhanced immune surveillance pressure. Conversely, the observed downregulation of PD-L1 during subsequent relapse treatment may be attributable to T cell depletion or dysfunction resulting from prolonged immune activation.

The determination of optimal administration sequence for oncolytic virotherapy in combination with chemotherapy and immunotherapy remains heterogeneous across clinical studies (29, 30). In this study, H101 was administered initially, with chemotherapy and immunotherapy subsequently administered the following day. Based on the remission of clinical symptoms and histopathological responses observed in the patient, we speculated that the first application of H101 can cause the lysis of tumor cells, release tumor-related antigens, activate antigen-presenting cells, and initiate the immune response of the body. At this time, the application of chemotherapy and immunotherapy drugs will greatly enhance the anti-tumor immune response of the body.

In conclusion, the combination therapy utilizing H101 offered substantial promise in the treatment of advanced EC, particularly for patients who were ineligible for standard therapy, and we can improve patient survival benefits by developing personalized treatment strategies. Despite reporting on only a single patient, our study still demonstrated the immunomodulatory effects of the combination therapy involving the H101 in individuals with recurrent advanced ESCC. This finding offers novel insights and potential directions for future clinical treatments. Nevertheless, the efficacy and safety of H101 combination therapy in advanced ESCC needs to be validated through large-scale clinical trials in the future. Above all, future study should include quantitative viral load analysis in lymph node specimens to provide definitive evidence of H101 replication and associated immune activation events. Furthermore, there is a pressing need to develop biomarkers for predicting combination therapy responses, investigate immunotherapy resistance mechanisms, and devise novel combination strategies.

## 4 Conclusion

We reported a case of successful reversal of immune resistance in advanced esophageal squamous cell carcinoma through intralymphatic injection of H101 combined with ICIs, and explore the mechanism of immunoresistance. The patient exhibited a significantly improved in PFS following initial administration of the H101 combination therapy. We also used a novel mode of administration, intralymphatic administration, which improved patient acceptance and convenience while achieving significant clinical efficacy.

## 5 Materials and methods

### 5.1 HE staining

The collected esophageal and cervical lymph node tissue specimens were sequentially processed through dehydration, paraffin embedding, and microtome sectioning. Following dewaxing, the sections were subjected to conventional hematoxylin and eosin (H&E) staining for histological examination under light microscopy.

### 5.2 Multiplex immunofluorescence

The multiplex immunofluorescence staining was performed on cervical lymph node tissue sections through sequential deparaffinization in xylene, rehydration in an ethanol gradient, and microwave-based antigen retrieval using EDTA buffer (pH 9.0). Subsequently, endogenous peroxidase blockade (5% H&_2_O&_2_) and serum blockade (10% rabbit serum or 3% BSA) were performed successively. The sequential labeling method was employed for six rounds of antigen detection: each round involved incubation with a specific primary antibody (1:1000, 1.5 h, room temperature), HRP-conjugated goat anti-mouse/rabbit universal secondary antibody (50 min), and TSA fluorescent dye (10 min). After each round of labeling, antibody elution was performed using microwave treatment with EDTA buffer (pH 9.0). Following DAPI counterstaining (10 min) and autofluorescence quenching, slides were mounted with antifade medium and imaged using a fluorescence microscope at specific excitation/emission wavelengths: 350/420 nm (DAPI), 490/520 nm (CD56), 550/570 nm (CD20), 590/620 nm (IL-1β), 630/690 nm (IFN-γ), 450/480 nm (CD206), and 750/780 nm (TCF-1).

## Data Availability

The original contributions presented in the study are included in the article/supplementary material. Further inquiries can be directed to the corresponding author.

## Supplementary material

Supplementary Figure 1 PET/CT scans of the cervical and axillary lymph nodes at first diagnosis and second recurrence. First diagnosis PET/CT revealed multiple enlarged lymph nodes with hypermetabolic activity throughout the body, and metastatic lesions were observed in the right cervical and left axillary lymph nodes. At the time of second recurrence, metastatic lymph nodes were detected in the right cervical region, mediastinum, hepatic hilum, and retroperitoneum.

Supplementary Figure 2 The positive rate of expression of immune-related factors. Between the two recurrences, there was an increasing trend in the infiltration of CD3, CD4, CD8, and CD20 immune cells in the tumor microenvironment after H101 injection, while PD-L1 and IFN-γ expression initially decreased and then increased, and IL-1β was consistently upregulated.
