# Supplementary figures and images for: Overcoming immune resistance in advanced esophageal squamous cell carcinoma with recombinant human adenovirus type 5 by impacting the immune microenvironment: a case report
Source: Front Immunol. 2025 Jun 30;16:1610058. doi: 10.3389/fimmu.2025.1610058 (PMC12256217; doi:10.3389/fimmu.2025.1610058)

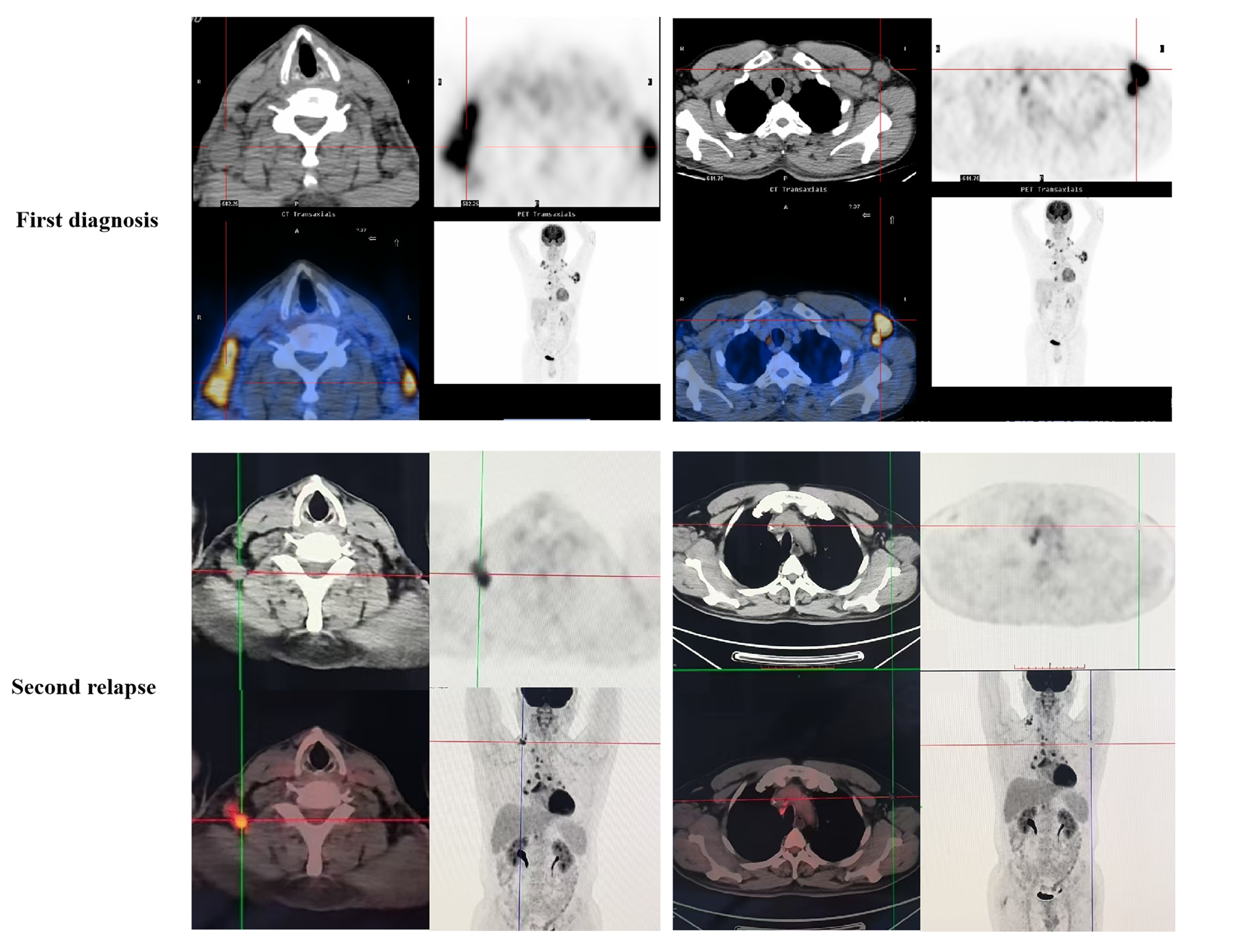

Supplement: Supplementary Figure 1 — PET/CT scans of the cervical and axillary lymph nodes at first diagnosis and second recurrence. First diagnosis PET/CT revealed multiple enlarged lymph nodes with hypermetabolic activity throughout the body, and metastatic lesions were observed in the right cervical and left axillary lymph nodes. At the time of second recurrence, metastatic lymph nodes were detected in the right cervical region, mediastinum, hepatic hilum, and retroperitoneum. [file Image1.tif]

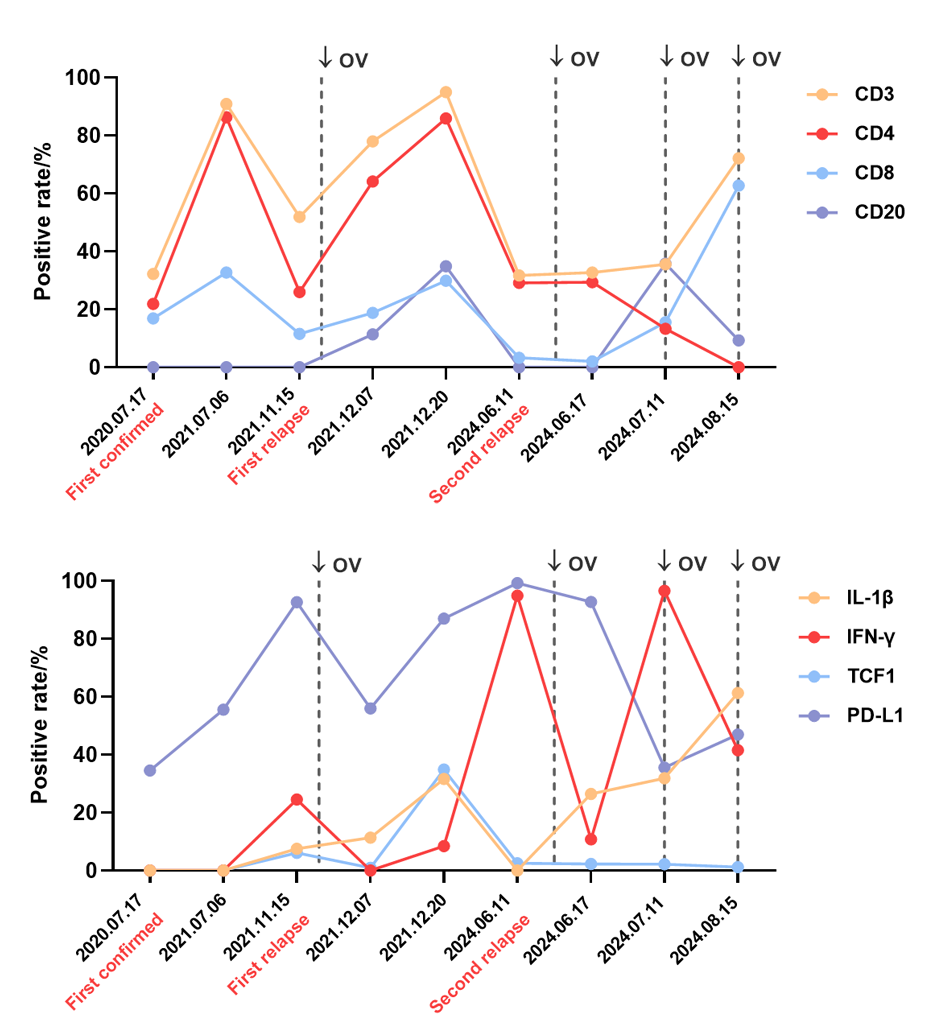

Supplement: Supplementary Figure 2 — The positive rate of expression of immune-related factors. Between the two recurrences, there was an increasing trend in the infiltration of CD3, CD4, CD8, and CD20 immune cells in the tumor microenvironment after H101 injection, while PD-L1 and IFN-γ expression initially decreased and then increased, and IL-1β was consistently upregulated. [file Image2.tif]
